# Triple-Divided Concha Bullosa: A New Anatomic Variation

**DOI:** 10.1155/2013/342615

**Published:** 2013-10-09

**Authors:** Turhan San, Barış Erdoğan, Bülent Taşel

**Affiliations:** Medeniyet University, Göztepe Education and Research Hospital, ENT Department, Eğitim Mah, Doktor Erkin Caddesi, Kadiköy, 34722 Istanbul, Turkey

## Abstract

In recent years, with the widespread use of imaging techniques such as paranasal sinus computed tomography (CT), many variations of nasal turbinates have been described. One of these variations known as concha bullosa (CB) is pneumatization of nasal turbinates. CB is the most frequently encountered anatomical variations of the middle turbinate. The term of septated concha bullosa has been described recently and it is an uncommon pneumatization anomaly of the middle turbinate. There has not been any study that correlates the number of septations and the presence of sinonasal pathologies. We hereby present a case of triple septated concha bullosa that has not been reported so far.

## 1. Introduction

Concha Bullosa (CB) is called partial or total pneumatization of the middle turbinate. CB is the most common anatomic variation of osteomeatal complex region that is generally seen in the middle turbinate, and rarely in the superior and inferior turbinate as well [1–3]. The exact reason of pneumatization of the middle turbinate is unknown. Although usually asymptomatic, an overpneumatized middle turbinate may constitute mass and in cases with impaired ventilation and drainage of osteomeatal region it can give rise to sinusitis. CB has first been described by Zuckerkandl in 1893 [4]. As far as we know, there is no publication related to triple divided CB in the literature. We report an uncommon anatomic variation in connection with concha bullosa pneumatization.

## 2. Case Report 

**Figure 1:**
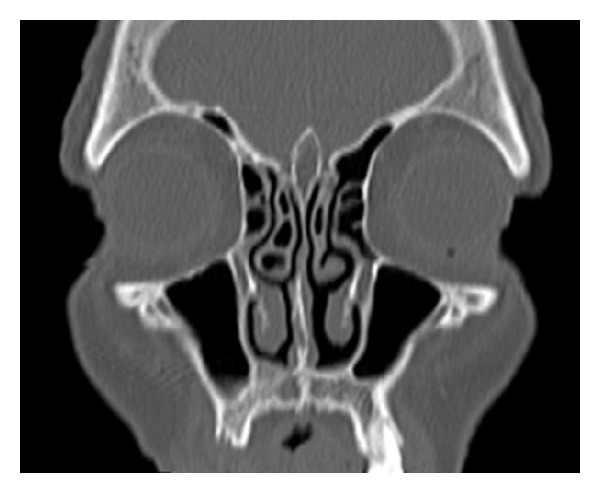
Coronal CT scan showing two thin bony septums inside the CB cavity and hypertrophy of inferior turbinate in the right side, besides lamellar type CB in the left side.

A-75-year old woman was admitted to our clinic with nasal obstruction and headache symptoms that had been going on for 10 years. In nasal anterior rhinoscopic and endoscopic examination, hypertrophy of the right middle turbinate and inferior turbinate were present. The patient suffered mostly from obstruction of right nasal cavity. Anterior rhinomanometry was performed after decongestion of the nose and a significant decrease in the nasal resistance was observed. A coronal plane CT demonstrated two thin bony septums inside the CB cavity and the hypertrophy of inferior turbinate in the right side and lamellar-type CB in the left side as well (Figure 1). Thus, CB cavity was divided into three cells. There was no attachment to the middle nasal meatus or sinus lateralis.

## 3. Discussion

The incidence of concha bullosa is 13–53%; anterior ethmoidal cells and posterior ethmoidal cells are responsible for pneumatization of CB approximately 55% and 45% of the cases, respectively [5, 6].

Pneumatization of the middle turbinate may originate from the frontal recess, anterior ethmoids, or directly from the middle meatus. Ventilation of the air cells within a concha bullosa comes from the area where the pneumatization originates [2].

Bolger et al. classified patients into three groups according to pneumatization degree of concha bullosa and localization [6]. These are lamellar, bullous, and extensive types. In the lamellar type, pneumatization is located in the vertical lamel of middle turbinate. In the bullous type, pneumatization is located in the inferior segment while in the extensive form all middle turbinate is pneumatized. The lamellar and bullous types are generally asymptomatic, while the extensive type associated with mechanical obstruction due to nasal blockage.

The severity of symptoms caused by concha bullosa is closely related to the degree of pneumatization. Especially, in cases with impaired ventilation and drainage of osteomeatal unit it can cause sinonasal pathologies. Some studies showed that CB may have a role in the etiology of chronic rhinosinusitis due to nasal blockage of the osteomeatal complex region, on the contrary some studies suggest that there is no statistically noticeably relationship between the presence of CB and rhinosinusitis [7].

Variations of the middle turbinate have been identified so far and have been reported. Some of these variations include: pneumatized, lateralized, hypoplastic, hypertrophic, paradoxically curved, secondary, accessory, bifurcate and trifurcate and septated middle turbinates [8–11]. Two cases of septated CB have been reported in the literature so far. Septated CB was first described by Yanagisawa et al. in 2008 [12]. The second case report was published by Perić et al. in 2009 [13]. These publications were about double septated CB. To our knowledge, triple septated CB has not been published in the rhinological literature yet. The middle turbinate is an important landmark of osteomeatal complex region, which is formed by the medial wall of the ethmoid sinus. It might originate from the frontal recess, middle meatus, or sinus lateralis. The middle turbinate is related to various functions of the nasal cavity, including olfaction, humidification, lubrication of the upper airways, regulation of airflow and temperature, and filtration [14].

The advancement of imaging techniques such as CT has provided us detailed information of patients' anatomy. This helps the surgeons to be aware of the anatomic variations in the osteomeatal region. Surgical resection of concha bullosa entails careful preservation of medial lamella (which attaches to the skull base) and resection of only lateral half of turbinate. The extent of middle turbinate pneumatization is evaluated on CT scans and this allows the surgeon to anticipate points of safe entry into the lumen of concha bullosa.

In our case, we present an uncommon anatomic variation of the middle turbinate which is called triple-septated concha bullosa. The surgeon must be aware of septations of the CB during the surgery because as the anatomy gets more complex, the likelihood of complications rises. Especially the anterior skull base will be vulnerable at the ethmoid roof during endoscopic surgery in such cases. Careful evaluation of CT scans before the surgery is essential in these cases. Its pathophysiology is unclear yet, and thus, there should be studies conducted with more cases. The patient refused surgery and thus histopathological examination was not performed.

## 4. Conclusion

Triple-septated concha bullosa is an uncommon anatomic variation of the middle turbinate. CT may easily identify such uncommon anatomic variation of the osteomeatal region. The studies with more cases are required for the better understanding of its histopathological structure.
